# Lipophilic Fraction of Cultivated *Bifurcaria bifurcata* R. Ross: Detailed Composition and In Vitro Prospection of Current Challenging Bioactive Properties

**DOI:** 10.3390/md15110340

**Published:** 2017-11-01

**Authors:** Sónia A. O. Santos, Stephanie S. Trindade, Catia S. D. Oliveira, Paula Parreira, Daniela Rosa, Maria F. Duarte, Isabel Ferreira, Maria T. Cruz, Andreia M. Rego, Maria H. Abreu, Silvia M. Rocha, Armando J. D. Silvestre

**Affiliations:** 1 CICECO—Aveiro Institute of Materials and Department of Chemistry, University of Aveiro, 3810-193 Aveiro, Portugal; sst@ua.pt (S.S.T.); cs.oliveira@ua.pt (C.S.D.O.); armsil@ua.pt (A.J.D.S.); 2 Centro de Biotecnologia Agrícola e Agro-Alimentar do Alentejo (CEBAL)/Instituto Politécnico de Beja (IPBeja), 7801-908 Beja, Portugal; parreira@i3s.up.pt (P.P.); daniela.rosa@cebal.pt (D.R.); fatima.duarte@cebal.pt (M.F.D.); 3 ICAAM—Instituto de Ciências Agrárias e Ambientais Mediterrânicas, Universidade de Évora, Pólo da Mitra, 7006-554 Évora, Portugal; 4 CNC—Center for Neuroscience and Cell Biology, University of Coimbra, 3004-504 Coimbra, Portugal; isabelcvf@gmail.com (I.F.); trosete@ff.uc.pt (M.T.C.); 5 FFUC—Faculty of Pharmacy, University of Coimbra, 3000-548 Coimbra, Portugal; 6 ALGAplus—Prod. e Comerc. De Algas e Seus Derivados, Lda., 3830-196 Ílhavo, Portugal; amrego@algaplus.pt (A.M.R.); htabreu@algaplus.pt (M.H.A.); 7 QOPNA and Department of Chemistry, University of Aveiro, 3810-193 Aveiro, Portugal; smrocha@ua.pt

**Keywords:** *Bifurcaria bifurcata*, macroalgae, lipids, lipophilic compounds, diterpenes, antioxidant activity, anti-inflammatory activity, antibacterial activity

## Abstract

Macroalgae have been seen as an alternative source of molecules with promising bioactivities to use in the prevention and treatment of current lifestyle diseases. In this vein, the lipophilic fraction of short-term (three weeks) cultivated *Bifurcaria bifurcata* was characterized in detail by gas chromatography–mass spectrometry (GC-MS). *B. bifurcata* dichloromethane extract was composed mainly by diterpenes (1892.78 ± 133.97 mg kg^−1^ dry weight (DW)), followed by fatty acids, both saturated (550.35 ± 15.67 mg kg^−1^ DW) and unsaturated (397.06 ± 18.44 mg kg^−1^ DW). Considerable amounts of sterols, namely fucosterol (317.68 ± 26.11 mg kg^−1^ DW) were also found. In vitro tests demonstrated that the *B. bifurcata* lipophilic extract show antioxidant, anti-inflammatory and antibacterial activities (against both Gram-positive and Gram-negative bacteria), using low extract concentrations (in the order of µg mL^−1^). Enhancement of antibiotic activity of drug families of major clinical importance was observed by the use of *B. bifurcata* extract. This enhancement of antibiotic activity depends on the microbial strain and on the antibiotic. This work represents the first detailed phytochemical study of the lipophilic extract of *B. bifurcata* and is, therefore, an important contribution for the valorization of *B. bifurcata* macroalgae, with promising applications in functional foods, nutraceutical, cosmetic and biomedical fields.

## 1. Introduction

Currently there is a remarkable demand for new bioactive molecules to treat diseases, caused by the modern lifestyle and the growing exposure to industrial pollutants and environmental toxins. Antioxidant compounds have been widely researched, due to their protective action against damage induced by oxidative stress, which is the major cause of diseases like cancer, diabetes, asthma, cardiovascular, or neurodegenerative problems [1], and of skin damage. Current lifestyle diseases have been also associated with inflammatory processes [2], which have also encouraged the scientific community to search for new molecules with anti-inflammatory capacity. At the same time, the emergence and spread of antibiotic-resistant bacteria strains is a growing public health problem [3], which has led the scientific community to search for new antibacterial substitutes or combination of drugs that might overcome this resistance [4]. 

Nature has always been an important source of molecules with biological properties as such, or after chemical modification [5]. In recent years, marine resources have become alternative sources of several value-added compounds [6,7]. Macroalgae, due to their high biological and chemical diversity and fast-growing properties, are one of the most explored marine resources [8,9]. In fact, approximately 28.5 million tons of macroalgae (brown, red or green) were produced worldwide in 2014, from both capture and aquaculture [10], with an estimated increase of 15%/year. This resource has been mainly explored for food consumption or for the production of specialty products, such as alginic acid, carrageenan, agar, or colorants [7]. In this context, the exploitation of macroalgae has been gaining increasing attention from several research groups around the world [11,12,13] and notably in Portugal [14,15,16,17,18]. In fact, the exploitation of marine resources is also one of the critical sectors for Portuguese development, given the extent and richness of its Exclusive Economic Zone (EEZ), being included in the Portuguese Research and Innovation Strategy for Smart Specialization for 2014–2020 [19].

Amongst the macroalgae species (close to 10,000), only a limited number have been the object of extensive studies due to their unique composition and consequent diversity of biological activities or health benefits [20]. Species belonging to the brown Dictyotaceae and Sargassaceae families have been studied in detail due to the presence of diterpenes, which have a wide range of biological properties, such as antimicrobial or antitumoral [21]. Among those families, *Bifurcaria bifurcata*, which can only be found in the northeastern Atlantic coasts, from Morocco to northwestern Ireland, is known to biosynthesize components rarely found in other macroalgae species, namely linear diterpenes [22]. In fact, the presence of these components in *B. bifurcata* has been widely reported [23,24,25,26,27]. In addition, several in vitro effects of *B. bifurcata* extracts, such as antimicrobial [28], antimitotic [29], or antiproliferative activity [30], have been attributed to the presence of diterpenes, although this assumption has never been adequately complemented by a detailed study of the composition of the tested extracts. In fact, the relationship between the chemical composition (limited to the fatty acids profile) and the biological properties (namely, antimicrobial and antioxidant activity) of *B. bifurcata* extracts has been addressed in only one study [31].

It has been suggested that the diterpenic composition of *B. bifurcata* is largely dependent on abiotic factors and especially on its geographic origin [32]. Furthermore, there are no studies regarding the lipophilic composition (and the diterpenic fraction in particular) of *B. bifurcata* cultivated in nutrient-rich waters (Integrated Multi-Trophic Aquaculture systems (IMTA)) (or even from the Portuguese coast), where, due to the totally different biotic and abiotic growth conditions, substantial differences in composition might be anticipated. 

In addition, most of the published studies using wild *B. bifurcata* have neglected the abundance of diterpenic components, and the few studies that reported their quantification have focused on specific fractions, not considering the lipophilic fraction as a whole [29,33,34]. The abundance of other important classes of lipophilic compounds are also unknown, namely sterols, long-chain aliphatic alcohols and even fatty acids, for which only a single study can be found [31]. The limited number of compounds identified in these studies can be overcome using gas chromatography coupled with mass spectrometry (GC-MS), which allows the detailed identification and quantification of complex mixtures of compounds found in the lipophilic extracts [35,36]. 

In this study the lipophilic fraction of *B. bifurcata* cultivated for a short period (three weeks) in a land-based IMTA system was detailed characterized by GC-MS analysis. In addition, in vitro assays were performed to evaluate the antioxidant, antibacterial and anti-inflammatory activities of this fraction. Experiments were also conducted aiming to access the synergistic antibiotic–*B. bifurcata* lipophilic extract bacterial growth inhibition by determining the minimum inhibitory concentration (MIC—the lowest concentration where no bacterial growth was detected).

## 2. Results and Discussion

### 2.1. Lipophilic Composition

The dichloromethane extraction yield of *B. bifurcata* accounted for 3.92 ± 0.09% (*w*/*w*), which is quite similar to the *n*-hexane extraction yield obtained for *B. bifurcata* from both aquaculture and wild collected in Portuguese coast [37]. Additionally, the dichloromethane extraction yield was considerably higher than those observed previously for other Phaeophyta species [15] or for Chlorophyta or Rhodophyta macroalgae [14].

The chemical composition of the *B. bifurcata* dichloromethane extract was studied in detail by GC-MS analysis. With the exception of the diterpenes family, all the lipophilic components were identified as their trimethylsilyl derivatives, after derivatization of the dichloromethane extract. The identification of the main lipophilic extractives and the corresponding quantification is summarized in Table 1. In general, the extract was mainly composed of diterpenes, free fatty acids (C14–C22), long-chain aliphatic alcohols (C14–C28), and sterols, among other components. To the best of our knowledge this is the first study reporting the analysis of the lipophilic fraction of *B. bifurcata* by GC-MS. Recently, Alves et al. [31] analyzed the dichloromethane extract of *B. bifurcata* by GC with flame ionization detector; however, only the presence of fatty acids was reported.

#### 2.1.1. Diterpenes and Other Terpenoids

**Figure 1:**
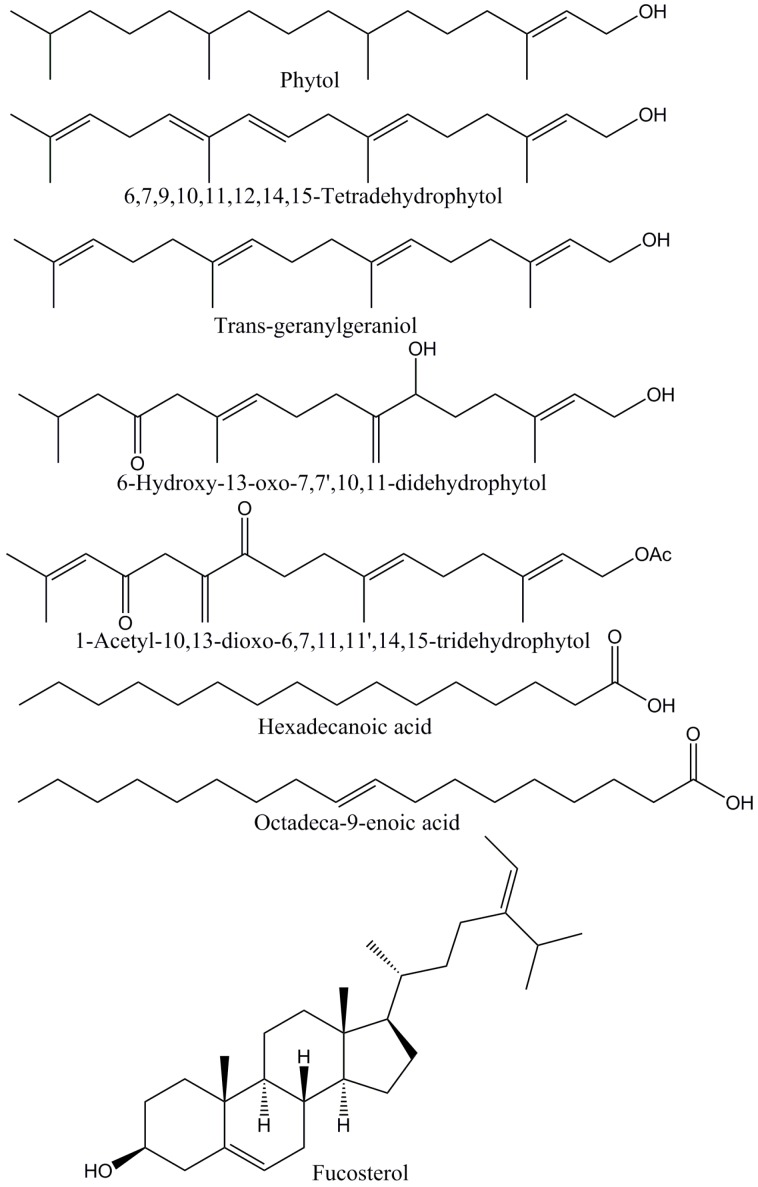
Diterpenes and other major lipophilic compounds detected in *B. bifurcata* dichloromethane extract.

Several diterpenes (Figure 1) and other terpenoids were identified in *B. bifurcata* dichloromethane extract, namely neophytadiene, phytol, *trans*-geranylgeraniol, 6,7,9,10,11,12,14,15-tetradehydrophytol, 6-hydroxy-13-oxo-7,7′,10,11-didehydrophytol, 1-acetyl-10,13-dioxo-6,7,11,11′,14,15-tridehydrophytol and 6,10,14-trimethyl-2-pentadecanone.

**Figure 2:**
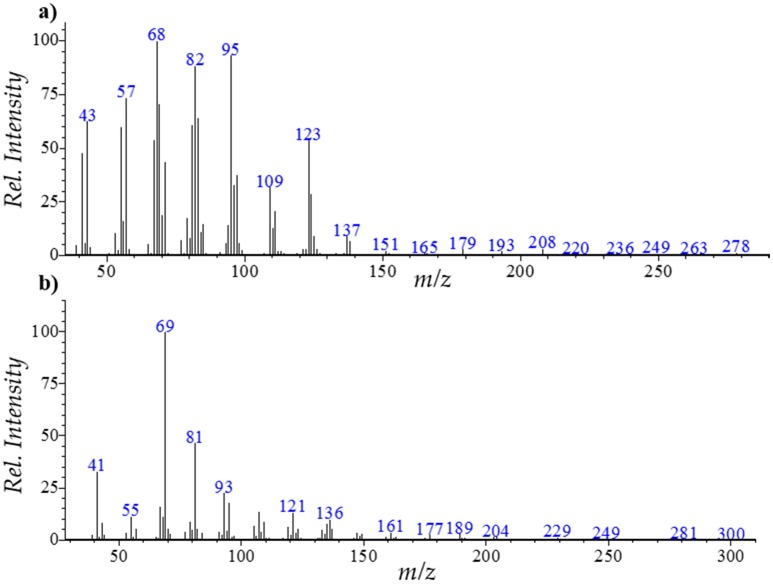
Mass spectra of (**a**) neophytadiene and (**b**) *trans*-geranylgeraniol.

The fragmentation patterns of these components depend on the number and position of the double bonds, and on the nature of the substituent groups [23,25,26]. As an example, the mass spectra of neophytadiene and *trans*-geranylgeraniol are presented in Figure 2. 

The mass spectrum of neophytadiene presents a molecular ion [M]^+^ at *m*/*z* 278 and major product ions at *m*/*z* 43 ([C_3_H_7_]^+^), 57 ([C_4_H_9_]^+^), 68, 82, 95 ([C_7_H_11_]^+^), 109 and 123 ([C_9_H_15_]^+^). Similarly, *trans*-geranylgeraniol presents a molecular ion [M]^+^ at *m*/*z* 290, and characteristic product ions at *m*/*z* 69 ([C_5_H_9_]^+^), 81 ([C_6_H_9_]^+^), 121 ([C_11_H_19_]^+^) and 272 (M-H_2_O]^+^). 

Diterpenes, accounting for 1892.78 ± 133.97 mg kg^−1^ DW, correspond to about 57% of the total amount of lipophilic compounds detected in *B. bifurcata* extract, with 1-acetyl-10,13-dioxo-6,7,11,11′,14,15-tridehydrophytol and 6-hydroxy-13-oxo-7,7′,10,11-didehydrophytol as the major components of this family, accounting for 1000.17 ± 94.47 and 637.84 ± 34.41 mg kg^−1^ DW, respectively. 1-acetyl-10,13-dioxo-6,7,11,11′,14,15-tridehydrophytol and 6-hydroxy-13-oxo-7,7′,10,11-didehydrophytol were already described as constituent of *B. bifurcata* collected in Brittany (France) [25,26], but no information was reported regarding its quantification. Considerable amounts of 6,7,9,10,11,12,14,15-tetradehydrophytol, *trans*-geranylgeraniol and neophytadiene were also found, and minor amounts of phytol were also detected. 6,7,9,10,11,12,14,15-Tetradehydrophytol was previously identified in *B. bifurcata* collected in Morocco [23], though no information has been provided concerning its abundance, while, to the best of our knowledge, phytol and neophytadiene are reported, for the first time, as constituents of *B. bifurcata*. In opposition, geranylgeraniol has been one of the most reported constituents of this macroalga species [26,29,38]. Additionally, the abundance of this component in the *B. bifurcata* dichloromethane extract is in the range of those (0.0–1.5 mg g^−1^ DW) reported in a seasonal variation study of the same species collected in Brittany (France) [39]. Notwithstanding, only traces of this diterpene were found in a geographical variation study of the diterpene composition of *B. bifurcata* collected in Spain [33]. 

Despite the high abundance verified for all the detected diterpenes, these were not reported in some of the studies regarding the seasonal and geographical variation of diterpenes in *B. bifurcata* [29,33,34].

Diterpenes have been associated with a wide number of health benefits [40]. In particular, those from macroalgae have been reported to have antioxidant, antimicrobial [41], antifungal [42], anti-viral [43], or antitumoral activities [44]. 

Minor amounts (10.79 ± 0.18 mg kg^−1^ DW) of 6,10,14-trimethyl-2-pentadecanone were found in *B. bifurcata* dichloromethane extract. This terpenoid and its derivatives have been described to be produced by macroalgae from Cystoseiraceae family, namely *Cystophora moniliformis* [45,46].

#### 2.1.2. Fatty Acids

Fatty acids were the second most abundant family of compounds detected in the lipophilic fraction of *B. bifurcata*, corresponding to 947.88 ± 21.94 mg kg^−1^ DW. Hexadecanoic acid (Figure 1) was the most abundant fatty acid (396.43 ± 17.01 mg kg^−1^ DW), followed by tetradecanoic (76.23 ± 1.69 mg kg^−1^ DW) and octadecanoic (41.01 ± 3.49 mg kg^−1^ DW) acids. The high abundance of hexadecanoic and tetradecanoic acid in *B. bifurcata* was already reported [31], however, in considerably higher amounts than those found in this study. 

Considerable amounts of unsaturated fatty acids were found in *B. bifurcata* (397.06 ± 18.44 mg kg^−1^ DW), representing about 42% of the total fatty acids. Octadec-9-enoic acid (Figure 1) was the most abundant unsaturated fatty acid, accounting for 200.95 ± 10.32 mg kg^−1^ DW, followed by the ω-3 octadeca-9,12,15-trienoic acid (57.81 ± 2.70 mg kg^−1^ DW) and the hexadec-9-enoic acid (42.24 ± 2.79 mg kg^−1^ DW). Significant amounts of the ω-3 fatty acid eicosa-5,8,11,14,17-pentaenoic acid were also found (35.86 ± 2.57 mg kg^−1^ DW), despite being 10 times lower than those reported previously by Alves et al. [31] for *B. bifurcata* collected in Peniche coast, Portugal. Notwithstanding, the high abundance of this component contributes to the low ω-6/ω-3 ratio verified for this macroalga (~0.46), which is considerably lower than the maximum (4:1) recommended for a diet in order to prevent the development of inflammatory processes [47]. Additionally, this ratio is significantly lower than those verified for other macroalgae species [15,48], which were considered promising to be incorporated in healthy diets. The high abundance of such components, widely associated with the prevention or delay of chronic diseases, such as cancer, cardiovascular, or coronary problems [49], highlights the potential of valorization of *B. bifurcata* also in food and/or nutraceutical industry. However, such exploitation requires that more detailed studies should be performed, namely evaluating their side effects and toxicity.

#### 2.1.3. Long-Chain Aliphatic Alcohols 

Minor amounts of long-chain aliphatic alcohols (LCAA) were found in *B. bifurcata* dichloromethane extract, accounting for 17.03 ± 1.29 mg kg^−1^ DW. To the best of our knowledge this is the first study reporting the presence of these components in *B. bifurcata*. Hexadecan-1-ol was the most abundant LCAA detected, followed by octadecan-1-ol and tetradecan-1-ol. 

#### 2.1.4. Sterols

Significant amounts of sterols were found in the dichloromethane extract of *B. bifurcata* (406.45 ± 26.19 mg kg^−1^ DW). Other studies reported the total sterols content in this macroalga collected in different countries [33,39]; however, only fucosterol has been described, with no further detailed characterization. In fact, fucosterol (Figure 1) was the major sterol detected in *B. bifurcata* dichloromethane extract, accounting for 317.68 ± 26.11 mg kg^−1^ DW. Notwithstanding, this value was lower than the previous reported contents (1400–5900 mg kg^−1^ DW) [33,39]. The bioactivities assigned to this phytosterol have been widely described in literature, such as its capacity to modulate cholesterol levels [50], its anti-inflammatory potential [51], or its dermo-protective effect [52]. Considerable amounts of desmosterol (43.56 ± 1.10 mg kg^−1^ DW) and campesterol (37.92 ± 1.61 mg kg^−1^ DW) were also found, which is in accordance with results observed for other Phaeophyta macroalgae [15]. Minor amounts of cholesterol were also detected.

#### 2.1.5. Monoglycerides

Monoglycerides were also detected in *B. bifurcata* dichloromethane extract, representing only a small fraction of the total lipophilic compounds identified (34.99 ± 1.10 mg kg^−1^ DW). To our knowledge, this is also the first study reporting the monoglycerides profile of *B. bifurcata*. 1-Monohexadecanoin was the most abundant compound detected from this family, representing almost 74% of the total monoglycerides. Minor amounts of 1-monooctadecenoin (5.17 ± 0.17 mg kg^−1^ DW) and 1-monoeicosa-tetraenoin (4.00 ± 0.31 mg kg^−1^ DW) were also found.

### 2.2. Antioxidant Activity 

The antioxidant activity of *B. bifurcata* dichloromethane extract was evaluated by both DPPH and ABTS in vitro assays. Table 2 presents the obtained results, expressed as the amount of extract needed to decrease the DPPH*^•^* and ABTS^+^*^•^* concentrations by 50% (IC_50_), as well as in mg of ascorbic acid (AAE) and trolox (TE) equivalents per g of dry weight. The IC_50_ values for ascorbic acid and BHT (for DPPH assay) and for trolox (for ABTS assay) were also estimated and are reported in Table 2 for comparative purposes. *B. bifurcata* lipophilic extract showed antioxidant activity against both radicals, although a higher activity was observed against ABTS^+^*^•^*, with the respective IC_50_ accounting for 116.25 ± 2.54 µg mL^−1^, representing 23.10 ± 0.51 mg of trolox equivalents g^−1^ DW.

To our knowledge, this is the first study reporting the antioxidant activity of a *B. bifurcata* extract against ABTS^+^*^•^*. In addition, the IC_50_ value determined for DPPH assay (365.57 ± 10.04 µg mL^−1^) was quite similar to that described before for a dichloromethane extract of *B. bifurcata* collected from rock pools in Portuguese coast [31]. Notwithstanding, *B. bifurcata* lipophilic extracts showed IC_50_ values against both radicals significantly lower than those determined for ascorbic acid or trolox (Table 2).

### 2.3. Anti-Inflammatory Activity

**Figure 3:**
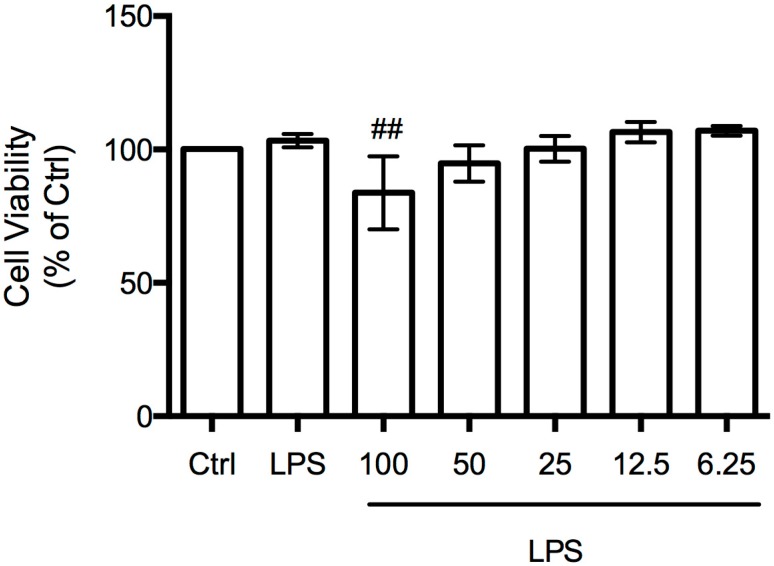
Effect of *B. bifurcata* dichloromethane extract at 6.25, 12.5, 25, 50, and 100 µg mL^−1^ on RAW 264.7 cell viability. Evaluation of statistical significance was performed using one-way ANOVA with *Dunnett’s* multiple comparison test; ## *p* < 0.01 compared to control (Ctrl).

In order to study the capacity of *B. bifurcata* dichloromethane extract to modulate nitric oxide production, an in vitro model of inflammation consisting of macrophages stimulated with LPS was performed. Concomitantly, cell viability was evaluated by the resazurin-based assay (Figure 3) in order to select concentrations with bioactivity and without cytotoxicity. *B. bifurcata* lipophilic extract showed a slightly but statistically significant cytotoxic effect at 100 µg mL^−1^, when compared to LPS-treated cells.

**Figure 4:**
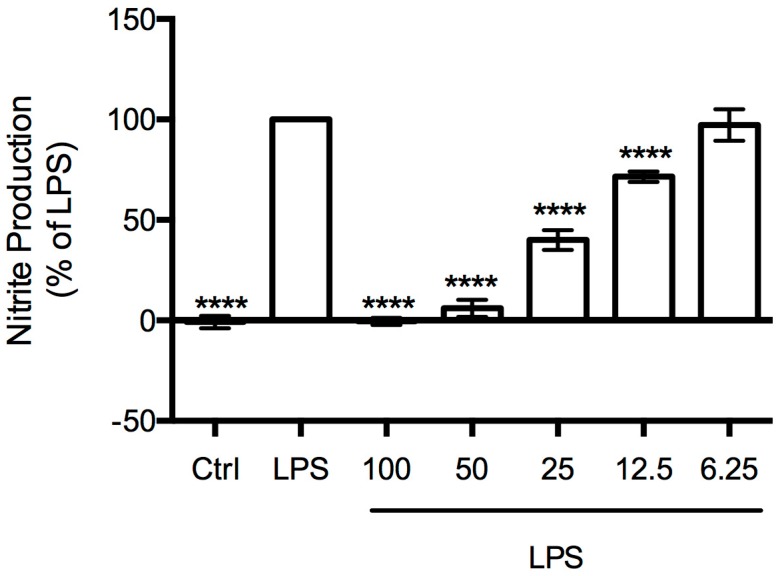
Inhibitory effect of *B. bifurcata* dichloromethane extract on nitric oxide production evoked by LPS in RAW 264.7 cells. *B. bifurcata* dichloromethane extract was used in the concentration range of 6.25–100 µg mL^−1^, **** *p* < 0.0001 compared to LPS.

The effect of *B. bifurcata* extract on NO production was analyzed by measuring the accumulation of nitrites in the culture medium, using the Griess assay. RAW 264.7 cells were stimulated with LPS in the presence or absence of *B. bifurcata* extract for 24 h. *B. bifurcata* extract inhibited LPS-induced NO production in a concentration-dependent manner (Figure 4). This extract markedly inhibited LPS-induced NO production to 6% and 40% at 50 and 25 µg mL^−1^, respectively.

The decrease in LPS-induced NO production at 50 µg mL^−1^ to levels similar to those observed in untreated cells reveals the remarkable anti-inflammatory potential of this extract. In fact, the anti-inflammatory activity of the extract is considerable higher than reported for other macroalgae extracts, namely from *Saccharina japonica* [53] or *Undaria pinnatifida* [54]. To our knowledge, this is the first study reporting the anti-inflammatory activity of *B. bifurcata*. 

### 2.4. Antibacterial Activity

The antibacterial activity of *B. bifurcata* lipophilic extract against *Staphylococcus aureus*, *Escherichiacoli*, *Pseudomonas aeruginosa*, and *Staphylococcus epidermidis* were evaluated. Therapeutic strategies to counteract infections caused by these bacterial strains have been one of the main concerns, particularly in health care settings. In fact, *S. aureus*, *E. coli*, and *P. aeruginosa* present the higher rates of antibiotic resistance, while *S. epidermidis* has been considered an opportunistic pathogen, being the most common source of infections on indwelling medical devices [55]. Thus, in this study, the antimicrobial efficacy of four antibiotics representing drug families of major clinical importance, such as aminoglycosides (Gent: Gentamicin), tetracyclines (Tetra: Tetracycline), macrocyclics (Rif: Rifampicin), and β-lactams antibiotics, such as aminopenicillins, (Amp: Ampicillin) were evaluated in combination with the *B. bifurcata* lipophilic extract.

Results regarding the antibacterial activity of the *B. bifurcata* dichloromethane extract are presented in Table 3. Activity against *S. aureus* ATCC^®^6538 (MIC = 1024 µg mL^−1^), *S. aureus* ATCC^®^43300 (MIC = 2048 µg mL^−1^) and *E. coli* (MIC = 2048 µg mL^−1^) was observed. No growth inhibition of *S. epidermidis* and *P. aeruginosa* PAO1 was verified in the range of concentrations tested (MIC > 2048 μg mL^−1^). *B. bifurcata* extract showed inhibition against both Gram-negative and Gram-positive bacteria, in opposition to that observed by Alves et al. [31], which only verified activity of a *B. bifurcata* dichloromethane extract against Gram-negative bacteria. Additionally, these authors have not reported growth inhibition against the same *E. coli* strain. Notwithstanding the fact that the extraction conditions in both studies were not the same, the macroalgae origins are distinct, which may alter the metabolite composition and therefore their bioactivities, highlighting the importance of controlling the growth conditions of macroalgae in order to maximize their valorization. 

The synergism of *B. bifurcata* dichloromethane extract with antibiotics was also evaluated against the same bacterial panel. This approach has been suggested as a promising tool to overcome the bacterial resistance that has been developed to most of the antibiotic classes recommended for their treatment [3,4,56]; to our knowledge, no similar study has previously been reported for this macroalga. The synergetic activity (antibiotic + extract) screening was performed against the bacterial strains for which a new antibiotic MIC was possible to determine, namely for *E. coli* ATCC^®^25922, *S. aureus* ATCC^®^43300 and *S. aureus* ATCC^®^6538 (Table 3). Interestingly, the combination of the extract with gentamicin or tetracycline drastically decreased the antibiotic MIC against the three strains under study, enabling it to be effective at considerably lower concentrations. Similar behavior was observed for both *E. coli* ATCC^®^25922 and *S. aureus* ATCC^®^43300 when the extract was combined with rifampicin. These observations suggest that the use of *B. bifurcata* lipophilic fraction may be considered within a coadjutant/synergistic approach to conventional antibiotherapy, enabling a superior therapeutic potential against the above-mentioned bacteria. 

Concerning the interaction between *B. bifurcata* dichloromethane extract and ampicillin, an antagonism effect was observed, with MIC values increasing considerably. This may be linked to alterations in the antibiotic structure induced by the presence of the extract in solution, leading to its partial inactivation. 

## 3. Material and Methods 

### 3.1. Sample

*Bifurcaria bifurcata* R. Ross was cultivated in the land-based aquaculture system of ALGAplus, Lda, at Ria de Aveiro (Portugal, 40°36′43″ N, 8°40′43″ W). The company operates under the IMTA concept, using solely filtered nutrient-rich water from a fishpond to produce seaweed biomass. The culture starting material was obtained at Aguda beach (Portugal, 41°2′38″ N, 8°39′10″ W) in May 2014 and grown by vegetative propagation during three weeks at constant conditions. The biomass was washed with seawater to remove salts, epiphytes, and/or microorganisms and dried at 25 °C until it reached a total moisture content of 12%. The samples were transformed into flakes (1–2 mm), packed, and stored in hermetic bags in the company’s storage room. 

### 3.2. Lipophilic Compounds Extraction

Three aliquots (20 g) of lyophilized macroalgae samples were Soxhlet extracted with dichloromethane (Sigma Chemical Co., Madrid, Spain) for 9 h. Solvent was evaporated to dryness, lipophilic extracts weighted and the results were expressed in percent of dry weight material (% DW). Dichloromethane was selected as a fairly specific solvent for lipophilic extractives isolation for analytical purposes only.

### 3.3. GC-MS Analysis

The analysis of the lipophilic extracts followed two distinct methodologies: the first suitable for trimethylsilyl (TMS) derivatizable compounds and the second for diterpenic compounds, as described below.

#### 3.3.1. Analysis of Trimethylsilyl Derivatizable Compounds

Before GC-MS analysis, two aliquots of each dried extract (20 mg each) and an accurate amount of internal standard (tetracosane, 0.25–0.50 mg, Sigma Chemical Co., Madrid, Spain) were dissolved in 250 μL of pyridine (Sigma Chemical Co.). 

The compounds containing hydroxyl and carboxyl groups were converted into TMS ethers and esters, respectively, by adding 250 μL of *N*,*O*-bis(trimethylsilyl)trifluoroacetamide (Sigma Chemical Co.) and 50 μL of trimethylchlorosilane (Sigma Chemical Co.), standing the mixture at 70 °C for 30 min [57]. The derivatized extracts were analyzed by GC-MS following previously described methodologies [15,35] on a GCMS-QP2010 Ultra (Shimadzu, Kyoto, Japan), equipped with a DB–1 J&W capillary column (30 m × 0.32 mm inner diameter, 0.25 μm film thickness). The chromatographic conditions were as follows: initial temperature, 80 °C for 5 min; temperature gradient, 4 °C min^−1^; final temperature, 260 °C; temperature gradient, 2 °C min^−1^; final temperature, 285 °C for 8 min; injector temperature, 250 °C; transfer-line temperature, 290 °C; split ratio, 1:33.

Compounds were identified as TMS derivatives by comparing their mass spectra with two commercial GC-MS spectral libraries (Wiley 275 and U.S. National Institute of Science and Technology (NIST14) ), their characteristic retention times obtained under the described experimental conditions [15,35], and by comparing their mass spectra fragmentation profiles with published data or by injection of standards.

For semi-quantitative analysis, GC-MS was calibrated with pure reference compounds, representative of the major lipophilic extractive families (cholesterol, hexadecanoic acid and nonadecan-1-ol (Sigma Chemical Co.)) relative to tetracosane. The respective response factors were calculated as an average of six GC-MS runs. Each one of the three extracts prepared from macroalgae was injected in duplicate (*n* = 6). The presented results are the average of the concordant values obtained (less than 5% variation between injections of the same aliquot and between triplicated extracts of the same macroalgae).

#### 3.3.2. Analysis of diterpenes

Diterpenes were identified and quantified by the analysis of the *B. bifurcata* extract without derivatization. About 10 mg of extract were dissolved in 1100 µL of dichloromethane with 0.8 mg of internal standard (*n*-hexadecane (Supelco, Bellefonte, PA, USA)).

The extracts were injected in the same GC-MS equipment as described above and the chromatographic conditions were as follows: initial temperature, 80 °C for 5 min; temperature gradient, 5 °C min^−1^, final temperature 200 °C, temperature gradient, 2 °C min^−1^, final temperature 240 °C; temperature gradient, 5 °C min^−1^, final temperature 285 °C for 8 min; injector temperature, 250 °C; transfer-line temperature, 290 °C; split ratio, 1:40. All other conditions were the same as described above. Diterpenes were identified by comparing their mass spectra fragmentation profile with library (Wiley 275 and U.S. National Institute of Science and Technology (NIST14)) and with the characteristic fragmentation pathway described in literature for these components [23,25,26].

Semi-quantitative analysis was carried out determining the response factor (an average of six GC-MS runs) of a representative standard, namely phytol (Sigma Chemical Co.), relative to *n*-hexadecane. Each one of the three aliquots were injected in duplicate (*n* = 6).

### 3.4. Antioxidant Activity

#### 3.4.1. DPPH Assay

The antioxidant activity of the *B. bifurcata* lipophilic extract was measured by their hydrogen-donating or radical scavenging ability using the stable free radical 2,2-diphenyl-1-picrylhydrazyl (DPPH*^•^*) (Sigma Chemical Co.), following a previously described procedure [35]: 0.25 mL of DPPH*^•^* (0.8 mM in methanol (Fluka Chemie, Madrid, Spain)) were added to 1.00 mL of the aqueous solution of the *B. bifurcata* dichloromethane extract and 2.75 mL of methanol. The extract concentration ranged between 61.9 and 556.9 μg mL^−1^. After 30 min of incubation in the dark, at room temperature, the absorbance was determined at 517 nm, using a Shimadzu UV-1800 spectrophotometer (Kyoto, Japan). Standards of ascorbic acid (Fluka Chemie) and 3,5-di-*tert*-4-butylhydroxytoluene (BHT) (Sigma Chemical Co. were used with concentrations ranging from 0.7 to 5.6 μg mL^−1^ and 5.0 to 75.8 μg mL^−1^. Duplicate measurements of three extracts were carried out (*n* = 6). The antioxidant activity was expressed in IC_50_ values, defined as the inhibitory concentration of the extract required to decrease the initial DPPH radical concentration by 50%, as well as in g of ascorbic acid equivalents per kg of dry weight (g AAE kg^−1^ DW).

#### 3.4.2. ABTS Assay

The ABTS assay is based on the scavenging of the 2,2′-azino-bis(3-ethylbenzothiazoline-6-sulphonic acid) radical cation, ABTS^+^*^•^* (Sigma Chemical Co.) converting it into a colorless product. In this test, ABTS^+^*^•^* cation was generated by reacting 50 mL of ABTS 7 mM solution with 25 mL of potassium persulfate (Fluka Chemie) 2.45 mM, following a methodology described before with minor modifications [35]. This mixture was then incubated in the dark, at room temperature, for 16 h. Before usage, the ABTS^+^*^•^* solution was diluted with methanol to obtain an absorbance of 0.700 ± 0.02 at 734 nm. 30 μL of *B. bifurcata* dichloromethane extract or trolox (Aldrich Chemical Co., Madrid, Spain), used as reference, were mixed with 3 mL of ABTS^+^*^•^* solution, obtaining final concentrations of 47.9–239.5 µg mL^−1^ and 1.0–5.0 µg mL^−1^, respectively. The absorbance was measured at 734 nm using a Shimadzu UV-1800 spectrophotometer (Kyoto, Japan). Duplicate measurements of three extracts were performed. 

The antioxidant activity was expressed as IC_50_ values (extract or trolox concentration providing 50% of ABTS^+^*^•^* inhibition) as well as in mg of trolox equivalents per g of dry weight (mg TE g^−1^ DW).

### 3.5. Anti-Inflammatory Activity

Test solutions of *B. bifurcata* dichloromethane extract (100 mg mL^−1^) were prepared in ethanol and diluted in a culture medium (prepared as described below). Ethanol concentrations ranged from 0.007 to 0.1% (*v*/*v*), corresponding to extract concentrations between 6.25 and 100 µg mL^−1^.

#### 3.5.1. Cell Culture

Raw 264.7, a mouse leukaemic monocyte macrophage cell line from American Type Culture Collection (ATCC TIB-71), kindly supplied by Dr. Otília Vieira (Centro de Neurociências e Biologia Celular, Universidade de Coimbra, Coimbra, Portugal), was cultured in Dulbecco’s Modified Eagle Medium supplemented with 10% non-inactivated fetal bovine serum, 100 U mL^−1^ penicillin, and 100 μg mL^−1^ streptomycin at 37 °C in a humidified atmosphere of 95% air and 5% CO_2_. During the experiments, cells were monitored by microscope observation in order to detect any morphological changes.

#### 3.5.2. Determination of Cell Viability

Assessment of metabolically active cells was performed using a resazurin (a nonfluorescent blue dye) based assay [58]. Briefly, cell duplicates were plated at a density of 0.1 × 10^6^/well, in a 96 well plate and allowed to stabilize overnight. Following this period, cells were either maintained in culture medium (control) or pre-incubated with *B. bifurcata* extract diluted in culture medium for 1 h, and later activated with 50 ng mL^−1^ of the *Toll-like* receptor 4 agonist lipopolysaccharide (LPS) for 24 h. After the treatments, resazurin solution (50 μM in culture medium) was added to each well and incubated at 37 °C for 1 h, in a humidified atmosphere of 95% air and 5% CO_2_. Viable cells are able to reduce resazurin into resorufin (fluorescent pink) and, hence, their number correlates with the magnitude of dye reduction. Quantification of resorufin was performed on a Biotek Synergy HT plate reader (Biotek, Winooski, VT, USA) at 570 nm, with a reference wavelength of 620 nm.

#### 3.5.3. Measurement of Nitrite Production by Griess Reagent

The production of nitric oxide (NO) was measured by the accumulation of nitrite in the culture supernatants of cells treated with or without *B. bifurcata* dichloromethane extract (6.25–100 µg mL^−1^) in the presence or absence of LPS, using a colorimetric reaction with the Griess reagent [59]. Briefly, 170 μL of culture supernatants were diluted with equal volumes of the Griess reagent [0.1% (*w*/*v*) *N*-(1-naphthyl)-ethylenediamine dihydrochloride and 1% (*w*/*v*) sulphanilamide containing 5% (*w*/*v*) H3PO4] and maintained during 30 min, in the dark. The absorbance at 550 nm was measured in a Biotek Synergy HT plate reader. Culture medium was used as blank and nitrite concentration was determined from a regression analysis using serial dilutions of sodium nitrite as standard (0.5–50 µM).

• Statistical Analysis

Statistical analysis was performed using GraphPad Prism 6.0 for Mac OS X (GraphPad Software, San Diego, CA, USA). For each experiment, the results are expressed as mean ± SD of, at least, three independent experiments. Evaluation of statistical significance was performed using one-way ANOVA with *Dunnett’s* multiple comparison test. Values of *p* < 0.05 were considered statistically significant.

### 3.6. Antibacterial Activity

#### 3.6.1. Bacterial Strains

Bacterial stocks of Gram-positive, *Staphylococcus aureus* ATCC^®^6538, *S. aureus* ATCC^®^43300 and *S. epidermidis* and Gram-negative *Escherichia coli* ATCC^®^25922 and *Pseudomonas aeruginosa* PAOI were kept in Brucella Broth (Fluka Chemie, Madrid, Spain) with 20% (*v*/*v*) glycerol (Sigma, Madrid, Spain) at −80 °C.

Pre-inoculum was prepared by stocks defrosting at room temperature, suspension in Mueller Hinton Broth (MHB; Liofilchem, Roseto degli Abruzzi, Italy) followed by a 6 h incubation at 37 °C, 220 rpm. Afterwards, bacteria were streaked onto Mueller Hinton Agar (MHA; Liofilchem, Roseto degli Abruzzi, Italy) and incubated overnight at 37 °C. Then, bacteria were harvested with sterile peptone water (Liofilchem), washed twice by centrifugation at 2700 rpm and bacterial pellet suspended in MHB. 

#### 3.6.2. Growth Kinetics

For each strain, growth curves were performed in three independent experiments in order to determine the respective exponential phase. Colonies from MHA were harvested with sterile peptone water and bacterial pre-inoculum was prepared as mentioned above. The initial optical density of each bacterial strain was adjusted to 0.04 in the referred media (λ = 600 nm). T-flasks (Starsted, Numbrecht, Germany) containing media and bacterial inoculums were incubated with agitation (220 rpm) at 37 °C. At different time points, samples were taken and the optical density was measured at λ = 600 nm. 

The correlation between the obtained optical density values with the number of colony-forming units per mL (CFU mL^−1^) was accessed. For CFU mL^−1^ determination, bacteria were harvested from liquid media at different time points; serial dilutions were done in peptone water (10^−2^ until 10^−7^) and 10 μL of each dilution plated in MHA. Incubation was done as previously described and the number of CFUs was determined after 24 h. Experiments were performed twice and in triplicate (*n* = 6 for each experiment). 

#### 3.6.3. Minimal Inhibitory Concentration (MIC) Determination

MIC assays were performed in accordance to the Clinical and Laboratory Standards Institute (CLSL) guidelines [60]. Briefly, bacterial cells in exponential growth phase (about 2 h of incubation, previously defined in Section 3.6.2) were suspended in MHB (Fluka Chemie) and the *B. bifurcata* dichloromethane extract was dissolved in dimethyl sulfoxide (DMSO; AppliChem, Darmstadt, Germany) to a final stock concentration of 50 mg mL^−1^. The antibacterial performance of the extract was screened using the microbroth dilution method in a range of concentrations from 8 to 2048 μg mL^−1^ in 96 well-plates (Starsted, Numbrecht, Germany) [61]. The MIC was qualitatively determined after 24 h of incubation, at 37 °C, by addition of 3-(4,5-dimethylthiazol-2-yl)-2,5-diphenyltetrazolium bromide (MTT, Calbiochem^®^ supplied by Millipore, Madrid, Spain) with slight adaptations to the protocol described by Ellof et al. [62]. The use of MTT, metabolized to a formazan chromophore by the viable cells, was required for MIC determination due to the coloration of the extract that turned the naked eye MIC visualization impracticable. The following controls were performed: (i) solvent control: bacterial cultures with 4% (*v*/*v*) of DMSO, which represents the maximum amount of solvent added to the cultures, to infer its effect on bacterial growth; (ii) pure cultures (only bacterial inoculum); and (iii) culture media. Experiments were performed twice and in triplicate (*n* = 6 for each experiment). 

#### 3.6.4. Synergistic Assays

The synergistic potential of the extract was accessed by conjugation of the extract with antibiotics, namely: rifampicin. gentamicin, ampicillin, and tetracycline (all supplied by Sigma, Madrid, Spain) in a concentration range between 2 and 512 µg mL^−1^; against the same bacterial panel. *B. bifurcata* extract was used at the respective MIC concentration, calculated as described above. Antibiotics MIC, when used alone or in extract + antibiotic combination were determined as mentioned above. Experiments were performed twice and in triplicate (*n* = 6 for each experiment).

## 4. Conclusions

The complete study of the lipophilic fraction of *B. bifurcata* from a Portuguese aquaculture system was characterized in detail by GC-MS. *B. bifurcata* dichloromethane extract was composed mainly of diterpenes (1892.78 ± 133.97 mg kg^−1^ DW), followed by fatty acids (947.88 ± 21.94 mg kg^−1^ DW). Considerable amounts of sterols (406.45 ± 26.19 mg kg^−1^ DW) were also found, with fucosterol accounting for 317.68 ± 26.11 mg kg^−1^ DW. The sterols, long-chain aliphatic alcohols, and monoglycerides profile of *B. bifurcata* is reported for the first time. Additionally, two diterpenes, phytol and neophytadiene, are also reported for the first time as constituents of *B. bifurcata*. This extract showed relevant antioxidant activities against both DPPH and ABTS radicals. Additionally, antibacterial activity was shown to be strain-dependent, with activity being verified against both Gram-positive (*S. aureus*) and Gram-negative (*E. coli*) strains. *B. bifurcata* extract was shown to increase antibiotic antimicrobial activity, with this effect being microbial strain- and antibiotic-dependent. This study provides the basis for further exploitation of this alga’s biomolecules for current antimicrobial chemotherapeutics, showing the practical utility of marine natural products to sensitize pathogenic bacteria, increasing their susceptibility. In addition, the studied extract showed promising anti-inflammatory activity, which is reported for the first time. It is important to point out that all biological activities were expressed using low extract concentrations, on the order of µg mL^−1^. This study is, therefore, an important contribution for the valorization of *B. bifurcata* macroalga from aquaculture systems, with promising applications in the functional food, nutraceutical, cosmetic, and biomedical fields, obviously considering the development of eco-friendly methodologies to extract this lipophilic fraction.

## Figures and Tables

**Table 1:** Compounds identified in *B. bifurcata* dichloromethane extract expressed in mg g^−1^ of extract and in mg kg^−1^ of dry macroalgae ^1^.

Rt (min)	Compound	mg g^−1^ of Extract	mg kg^−1^ of Dry Macroalgae
	**Fatty acids ^2^**	**24.18 ± 0.56**	**947.88 ± 21.94**
	*Saturated*	14.04 ± 0.40	550.35 ± 15.67
31.0	Tetradecanoic acid	1.94 ± 0.04	76.23 ± 1.69
33.5	Pentadecanoic acid	0.24 ± 0.02	9.45 ± 0.60
36.0	Hexadecanoic acid	10.11 ± 0.43	396.43 ± 17.01
38.2	Heptadecanoic acid	0.30 ± 0.02	11.81 ± 0.78
40.5	Octadecanoic acid	1.05 ± 0.09	41.01 ± 3.49
44.7	Docosanoic acid	0.24 ± 0.01	9.28 ± 0.48
48.5	Tetracosanoic acid	0.16 ± 0.01	6.15 ± 0.20
	*Unsaturated*	10.13 ± 0.47	397.06 ± 18.44
35.3	Hexadec-9-enoic acid	1.08 ± 0.07	42.24 ± 2.79
39.5	Octadeca-9,12-dienoic acid	1.04 ± 0.05	40.74 ± 2.08
39.6	Octadeca-9,12,15-trienoic acid	1.47 ± 0.07	57.81 ± 2.70
39.9	Octadec-9-enoic acid	5.13 ± 0.26	200.95 ± 10.32
42.9	Eicosa-5,8,11,14,17-pentaenoic acid	0.91 ± 0.07	35.86 ± 2.57
43.3	Eicosa-5,8,11-trienoic acid	0.02 ± 0.00	0.75 ± 0.03
43.4	Eicosa-11,14-dienoic acid	0.07 ± 0.01	2.86 ± 0.05
	*ω-hydroxyacids*	0.01 ± 0.00	0.47 ± 0.02
55.3	22-Hydroxydocosanoic acid	0.01 ± 0.00	0.47 ± 0.02
	**Long-chain aliphatic alcohols ^2^**	**0.43 ± 0.03**	**17.03 ± 1.29**
29.0	Tetradecan-1-ol	0.10 ± 0.00	3.78 ± 0.02
34.1	Hexadecan-1-ol	0.15 ± 0.01	5.89 ± 0.38
38.7	Octadecan-1-ol	0.09 ± 0.00	3.41 ± 0.19
58.9	Octacosan-1-ol	0.12 ± 0.00	4.78 ± 0.17
	**Sterols ^2^**	**10.37 ± 0.67**	**406.45 ± 26.19**
58.6	Cholesterol	0.19 ± 0.01	7.29 ± 0.24
62.0	Desmosterol	1.11 ± 0.03	43.56 ± 1.10
63.1	Fucosterol	8.10 ± 0.67	317.68 ± 26.11
63.4	Campesterol	0.97 ± 0.04	37.92 ± 1.61
	**Monoglycerides ^2^**	**0.89 ± 0.03**	**34.99 ± 1.10**
47.8	1-Monohexadecanoin	0.66 ± 0.03	25.82 ± 1.01
50.9	1-Monooctadecenoin	0.13 ± 0.00	5.17 ± 0.17
51.5	1-Monoeicosa-tetraenoin	0.10 ± 0.01	4.00 ± 0.31
	**Diterpenes ^3^**	**48.29 ± 3.42**	**1892.78 ± 133.97**
26.8	Neophytadiene	1.53 ± 0.08	59.86 ± 3.29
32.4	Phytol	0.87 ± 0.07	34.02 ± 3.25
34.0	*Trans*-geranylgeraniol	1.70 ± 0.16	66.53 ± 2.93
34.5	6,7,9,10,11,12,14,15-Tetradehydrophytol	2.41 ± 0.88	94.35 ± 6.29
36.5	6-Hydroxy-13-oxo-7,7′,10,11-didehydrophytol	16.27 ± 2.41	637.84 ± 34.41
40.7	1-Acetyl-10,13-dioxo-6,7,11,11′,14,15-tridehydrophytol	25.51 ± 4.04	1000.17 ± 94.47
	**Other terpenic compounds ^3^**	**0.28 ± 0.00**	**10.79 ± 0.18**
26.6	6,10,14-Trimethyl-2-pentadecanone	0.28 ± 0.00	10.79 ± 0.18
	**Total**	**84.44 ± 4.14**	**3309.93 ± 162.25**

^1^ Results are the average of the concordant values obtained from the triplicated extracts each injected twice (less than 5% variation between injections of the same aliquot and between triplicated dichloromethane extracts); ^2^ Compounds identified as trimethylsilyl derivatives; ^3^ Compounds identified in the method without derivatization.

**Table 2:** Antioxidant activity of *B. bifurcata* dichloromethane extract expressed as IC_50_ values (µg mL^−1^) and in mg of ascorbic acid (AAE)/trolox equivalents (TE) g^−1^ of dry weight.

Heading	DPPH Assay		ABTS Assay	
	IC_50_	mg AAE g^−1^ DW	IC_50_	mg TE g^−1^ DW
*B. bifurcata*	365.57 ± 10.04	11.18 ± 0.30	116.25 ± 2.54	23.10 ± 0.51
Ascorbic acid	4.08 ± 0.05			
BHT	14.32 ± 0.69			
Trolox			2.68 ± 0.07	

**Table 3:** Antibacterial activity of *B. bifurcata* dichloromethane extract and synergistic effects with different antibiotics expressed in MIC (µg mL^−1^).

Bacteria	Ext	Rif	Rif+ Ext	Gent	Gent+ Ext	Amp	Amp+ Ext	Tetra	Tetra + Ext
	MIC (µg mL^−1^)								
*E. coli* ATCC^®^25922	2048	32	16	>256	<2	32	128	18	<2
*Staphylococcus aureus* ATCC^®^43300	2048	16	<2	>256	16	128	256	>256	<2
*Staphylococcus aureus* ATCC^®^6538	1024	64	64	32	16	256	512	16	8
*Pseudomonas aeruginosa* PAO1	>2048	32	-	2	-	16	-	16	-
*Staphylococcus epidermidis*	>2048	32	-	512	-	>2048	-	32	-

Key: Ext: Extract; Rif: Rifampicin; Gent: Gentamicin; Amp: Ampicillin; Tetra: Tetracycline.
